# Significant glial alterations in response to iron loading in a novel organotypic hippocampal slice culture model

**DOI:** 10.1038/srep36410

**Published:** 2016-11-03

**Authors:** Sinead Healy, Jill McMahon, Peter Owens, Una FitzGerald

**Affiliations:** 1 Galway Neuroscience Centre, School of Natural Sciences, National University of Ireland, Galway, Ireland; 2 Centre for Microscopy and Imaging, National University of Ireland, Galway, Ireland

## Abstract

Aberrant iron deposition in the brain is associated with neurodegenerative disorders including Multiple Sclerosis, Alzheimer’s disease and Parkinson’s disease. To study the collective response to iron loading, we have used hippocampal organotypic slices as a platform to develop a novel *ex vivo* model of iron accumulation. We demonstrated differential uptake and toxicity of iron after 12 h exposure to 10 μM ferrous ammonium sulphate, ferric citrate or ferrocene. Having established the supremacy of ferrocene in this model, the cultures were then loaded with 0.1–100 μM ferrocene for 12 h. One μM ferrocene exposure produced the maximal 1.6-fold increase in iron compared with vehicle. This was accompanied by a 1.4-fold increase in ferritin transcripts and mild toxicity. Using dual-immunohistochemistry, we detected ferritin in oligodendrocytes, microglia, but rarely in astrocytes and never in neurons in iron-loaded slice cultures. Moreover, iron loading led to a 15% loss of olig2-positive cells and a 16% increase in number and greater activation of microglia compared with vehicle. However, there was no appreciable effect of iron loading on astrocytes. In what we believe is a significant advance on traditional mono- or dual-cultures, our novel *ex vivo* slice-culture model allows characterization of the collective response of brain cells to iron-loading.

Iron is indispensable for normal CNS function, being a crucial cofactor for enzymes involved in neurotransmitter synthesis, energy metabolism, myelin production, oxygen transport, DNA synthesis and repair and respiratory activity^1^. However, the redox-active nature of iron means that it can generate free radicals via the Fenton reaction and cause tissue damage if not properly regulated. Its metabolism, therefore, requires a sophisticated control system to minimize the potential deleterious effects of iron without compromising its availability for necessary cellular functions.

Neurons, astrocytes, microglia and oligodendrocytes are all equipped with different sets of iron-related molecules responsible for uptake, storage, use and export of iron^1^. The amount of each protein expressed varies greatly depending on the cell type and its iron status, brain region, developmental age, detection method and species. Briefly, iron influx into cells is controlled primarily by the transferrin receptor 1 (TfR1) and the divalent metal transporter 1 (DMT1). Excess iron is stored in a ferritin shell, comprised of heavy- and/or light-chain subunits or mitochondrial ferritin (FTH, FTL, FtMt). Ferroportin, which is the only known iron exporter, releases only ferrous iron. It is assisted in this function by the ferroxidases hephaestin and/or ceruloplasmin whose roles include production of ferric iron from the ferrous form and stabilization of ferroportin at the plasma membrane. Hepcidin prevents iron release via this route by causing internalization and degradation of ferroportin. This tightly-regulated system for handling iron can deteriorate or become overwhelmed and this might contribute to disease pathogenesis.

Iron is known to accumulate in the brains of healthy people with age^2^,^3^ and aberrant iron deposition in the brain has been reported (along with other commonalities such as inflammation and microglial activation) in a number of neurological disorders including Multiple Sclerosis^3^,^4^, Amyotrophic Lateral Sclerosis^5^,^6^, Alzheimer’s disease^1^,^7^, Parkinson’s disease^1^,^6^,^7^ and Huntington’s disease^1^,^5^,^7^. For instance, hephaestin (Heph) and ceruloplasmin (Cp) were shown to be upregulated in oligodendrocytes and astrocytes near inflamed lesion edges in post-mortem Multiple Sclerosis brain tissue^3^. Also, mutations in the genes encoding proteins involved in iron homeostasis, such as ceruloplasmin and L-ferritin, are known to cause neurodegeneration with brain iron accumulation^5^. However, to date, despite the heightened research interest in the endogenous iron handling system, it remains unclear whether metabolic dyshomeostasis of iron is causative or is a consequence of brain pathology.

Although MRI scanning can provide information on the location of iron deposits in brain and histological analyses can be carried out on post-mortem brain samples, neither of these methodologies can be used to provide detailed information on inter- and intra-cellular iron movement or on the cellular basis of iron-induced pathology. By using *in vitro* models, however, it is possible to study iron movement in relation to putative injury processes within a controlled, convenient and low-cost environment^6^,^8^,^9^,^10^,^11^,^12^,^13^.

Of these models, the most popular and convenient are monocultures and these have yielded much valuable information regarding iron metabolism, storage and transport in neurons and glial cells. However, this convenience comes at the cost of revealing an incomplete picture of the iron handling system. Several studies have shown that different brain cell types work in concert with respect to iron uptake and transport e.g. neurons co-cultured with microglia will upregulate hepcidin (a master regulator of iron homeostasis) in response to LPS exposure, but this effect does not occur in neuronal monocultures^14^. Similarly, despite reports of the dramatic uptake of iron nanoparticles in monocultures of astrocytes, neurons and oligodendrocytes^15^,^16^, these findings were not replicated in mixed dispersed cultures or organotypic slices. Instead, the nanoparticles were predominantly taken up by microglia and uptake by other cells remained modest.

Such findings highlight the necessity of using a more appropriate model so that the iron handling system in the brain may be properly studied and understood. Organotypic cultures are efficient and reliable *ex vivo* models, having the accessibility and convenience of *in vitro* models while preserving the complex *in vivo* brain milieu^17^,^18^. As such, they offer an ideal and, to our knowledge, novel platform for iron loading studies.

In the present study, we have developed a rat hippocampal slice culture model that uses iron loading to promote cellular iron accumulation. The iron loading reagents ferrocene, ferrous ammonium sulphate (FAS) and ferric citrate (FC) were tested and compared and the model was validated by comparisons with neonatal rat brain tissue harvested at equivalent time-points. The effect of iron loading on hippocampal oligodendrocytes, microglia, astrocytes and neurons was determined.

## Results

### Levels of iron in cultured hippocampal slices are representative of those found *in vivo*

**Figure 1:**
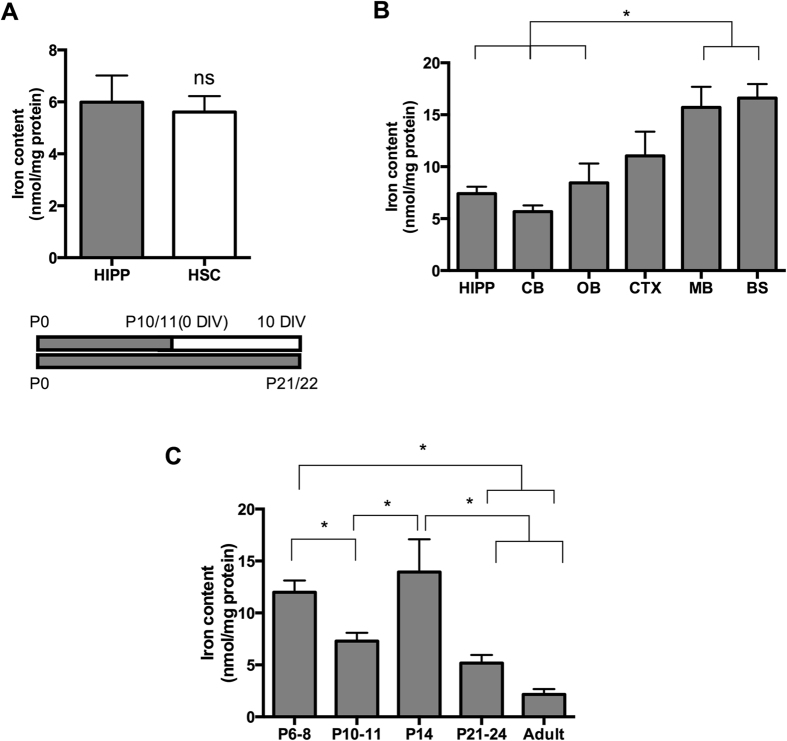
Validation of Organotypic Hippocampal Model. (**A**) *In vivo* (HIPP; P21) and *ex vivo* (HSC; P10/11 + 10DIV) hippocampal iron content do not differ significantly (assessed by unpaired t-test). (**B**) Iron content in postnatal brain (P10/11; N = 6 for each group). Hippocampal iron content matches levels of iron detected in cerebellum (CB), olfactory bulb (OB) and cortex (CTX) but is lower than the midbrain (MB) and brainstem (BS) at P10/P11 (**C**). There is a significant difference in iron content during neonatal hippocampal development. *Denotes P < 0.05 and **P < 0.01.

In order to determine if levels of iron in cultured slices reflect those present in age-matched tissue *in vivo*, the amount of iron in *ex vivo* P10/P11-harvested hippocampal slices that had been cultured for 10 days *in vitro* (DIV) was compared to the level of iron detected in hippocampal dissectates isolated from P21 rats. Tissue from P21 animals had 5.99 ± 1.03 nmol iron/mg protein, which did not differ significantly from the 4.97 ± 0.57 nmol/mg protein of P10/P11 +10 DIV cultured slice tissue (Fig. 1A).

To discover how levels of iron in rat hippocampus *in vivo* compared to those in other brain regions, quantities of iron were measured in dissectates from the hippocampus, olfactory bulb, cerebellum, cortex, brainstem and midbrain (Fig. 1B). Hippocampal iron content was 7.3 ± 0.67 nmol/mg, which did not differ significantly from iron content in the cerebellum (5.7 ± 0.60 nmol/mg), cortex (11 ± 2.3 nmol/mg) or olfactory bulb (8.4 ± 1.9 nmol/mg). However, iron content was significantly higher in the brain stem (17 ± 1.3 nmol/mg) and midbrain (MB;16 ± 2 nmol/mg) when compared to the hippocampus (P < 0.01). Moreover, a decrease in iron content was observed in the maturing postnatal hippocampus *in vivo* (aged P6-45; Fig. 1C; P < 0.05).

### Ferrocene exposure leads to maximal accumulation of iron

**Figure 2:**
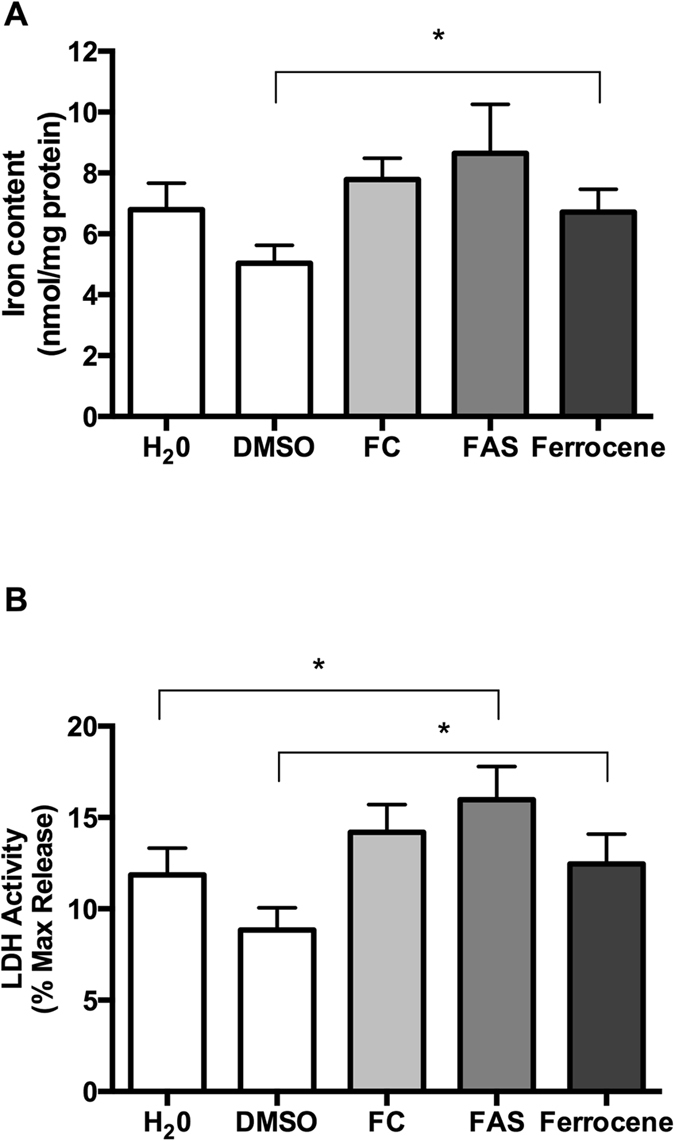
Iron reagent comparison. Cultures were treated for 12 h with 10 μM ferrocene, FAS or FC. (**A**) Iron Content. Ferrocene produced a significant 1.3-fold increase in iron content compared with DMSO (8.05 ± 0.98 and 4.97 ± 0.57, respectively). (**B**) Viability. Delivery of both FAS and ferrocene resulted in impaired viability whereas FC had no effect. *P < 0.05, **P < 0.01 compared with vehicle.

The composition of CSF is considered to reflect that of the interstitial fluid, which bathes neurons and glia^19^,^20^,^21^,^22^. With this in mind, and given that normal CSF contains 0.2–1.2 μM iron, cultures were supplemented with 10 μM iron reagent, as a means of mimicking iron overloading. Ten μM ferrocene, ferrous ammonium sulphate (FAS) or ferric citrate (FC) was added to cultured slices for 12 h in the absence of serum. Ferrocene produced a significant 1.3-fold increase in the levels of iron in slice cultures when compared with vehicle (P < 0.05; Fig. 2A). This ferrocene-induced increase in iron content was accompanied by minor toxicity, as indicated by a 1.4-fold increase in LDH release compared with vehicle (P < 0.05; Fig. 2B). By comparison, FAS and FC produced only a 1.2-fold or no increase in iron content (Fig. 2A,B). Nevertheless, FAS loading also caused a significant 1.3-fold increase in LDH activity, whereas FC had no effect (Fig. 2B). There was no significant difference in protein content between cells incubated with iron reagents compared with vehicle (data not shown). This experiment was repeated using 1 μM ferrocene, 1 μM FAS and 1 μM FC and, once again, the use of ferrocene was shown to result in a significant increase in brain slice iron levels (Fig. S1).

**Figure 3:**
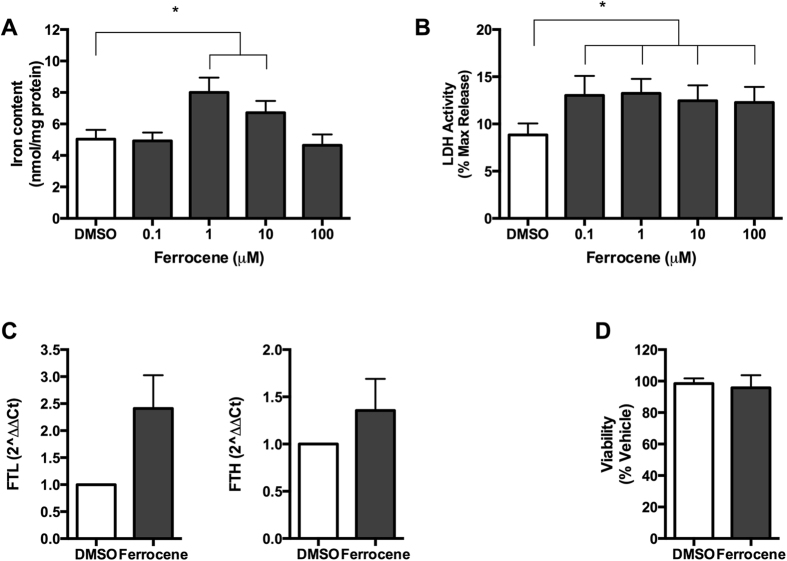
One μM ferrocene produced maximal iron accumulation. Cultures were loaded with 0.1–100 μM ferrocene for 12 h to identify optimal concentration for iron loading. (**A**) Iron Content. One μM ferrocene produced a 1.6-fold increase in iron content compared with DMSO vehicle (8.05 ± 0.98 and 4.97 ± 0.57 nmol/mg, respectively). (**B**) LDH Assay. All concentrations of ferrocene resulted in impaired viability, as determined by LDH release. (**C**) One μM ferrocene, the maximal iron loading concentration, produced a 2.4- and 1.4-fold up-regulation in Ferritin Light Chain (FTL) and Ferritin Heavy Chain (FTH) transcripts, respectively, (ferritin, an iron storage protein, is a surrogate indicator of iron accumulation) expression. (**D**) MTT. *Denotes P < 0.05 compared with vehicle.

Having established the supremacy of 10 μM ferrocene over the same concentration of FC or FAS in this model, a dose-response experiment was completed by testing the effects on cultured hippocampal slices of a 12-hour exposure to between 0.1 and 100 μM ferrocene. Ferrocene loading at 1 and 10 μM produced significant increases in iron content (P < 0.05; Fig. 3A). One μM ferrocene produced a maximal 1.6-fold increase in iron content compared with vehicle (8.05 ± 0.98 and 4.97 ± 0.57 nmol/mg, respectively) and 1.2-fold increase compared with 10 μM ferrocene. Using an LDH assay, we showed that all ferrocene concentrations tested here increased LDH activity by approximately 1.3-fold (P < 0.05; Fig. 3B).

To confirm iron accumulation, we also assessed the expression of ferritin light chain and ferritin heavy chain (i.e. FTL and FTH; surrogate markers for iron accumulation) in 1 μM ferrocene-loaded cultures. We found a 2.4-fold increase in FTL and 1.4-fold increase in FTH transcripts compared with vehicle-treated cultures (Fig. 3C). Using an MTT assay, we also measured metabolic activity of cultures exposed to 1 μM ferrocene. In contrast to the LDH viability data, no iron-induced loss in metabolic activity was detected (Fig. 3D).

### Ferritin accumulates in oligodendrocytes and microglia

Ferritin, an iron storage molecule that has been shown to be increased with increased iron accumulation, was used as a surrogate marker for iron to further determine the cellular localisation of the iron accumulation.

**Figure 4:**
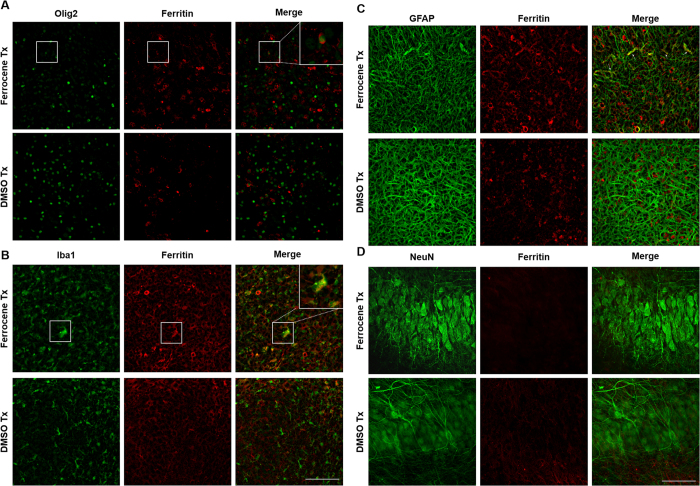
Ferritin expression in CNS cells in vehicle and iron-loaded slice cultures. Confocal images of (**A**) oligodendrocytes, (**B**) microglia, (**C**) astrocytes and (**D**) neurons. Insets show an enlarged version of the boxed area. Scale = 100 μm.

Double immunofluorescence labelling with cell type-specific antibodies and an antibody to ferritin showed ferritin expression in oligodendrocytes and microglia once they were treated with 1 μM ferrocene (Fig. 4A,B). In contrast, ferritin expression was rarely detectable in astrocytes and was not detectable in neurons (Fig. 4C,D). In control conditions, ferritin immunostaining was found at low levels predominantly in oligodendrocytes and, to a lesser extent, microglia. However, astrocytes and neurons did not have detectable ferritin under any culture conditions examined.

### Iron loading alters number and morphology of glial cells

**Figure 5:**
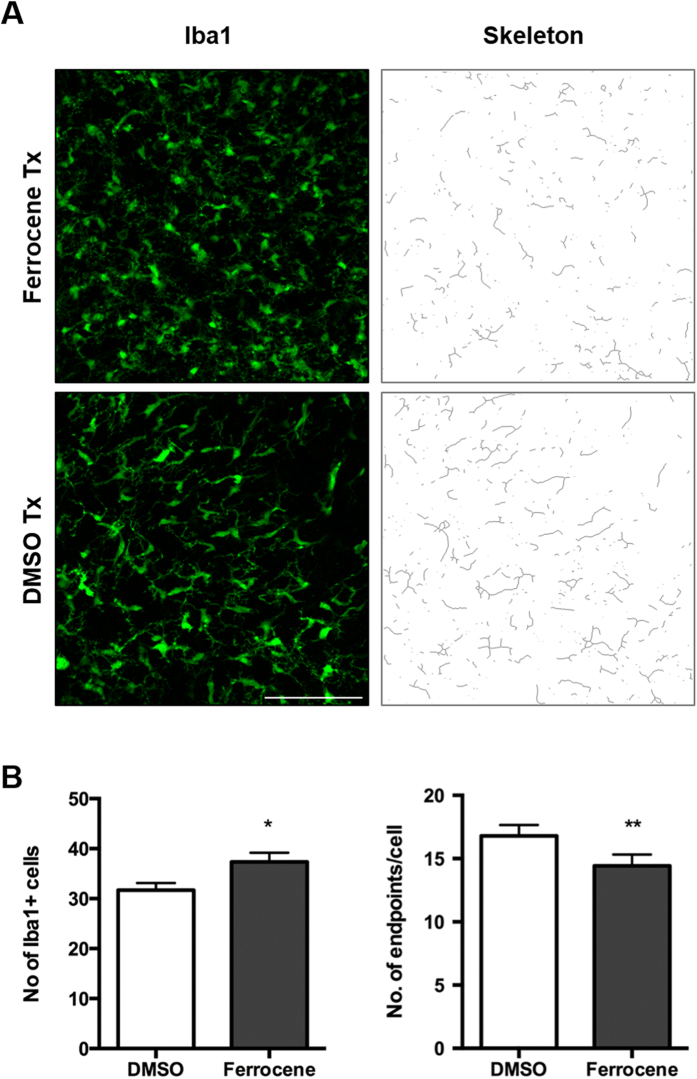
Effect of 1 μM ferrocene loading on the number and morphology of microglia. Representative confocal maximum intensity projection images of microglia and skeletonised images. (**B**) Quantification of the number of the number and branching complexity of IBA1-positive microglia per field. Iron loading increases number of microglia and reduces microglia branching complexity. *Denotes P < 0.05 and **P < 0.01 compared with vehicle. Scale = 100 μm.

Aside from the occurrence of cellular iron accumulation, iron loading with ferrocene also affected glial morphology and number. A significant 15% increase in the number of Iba1-positive microglia was observed (Fig. 5A,B). These cells also showed decreased complexity in branching morphology, as quantified by skeleton analysis, with the number of microglia endpoints per cell decreasing by 17% from 17 ± 0.86 in vehicle-treated slice cultures to 14 ± 0.9 in iron-loaded slice cultures (Figs 5 and S2).

**Figure 6:**
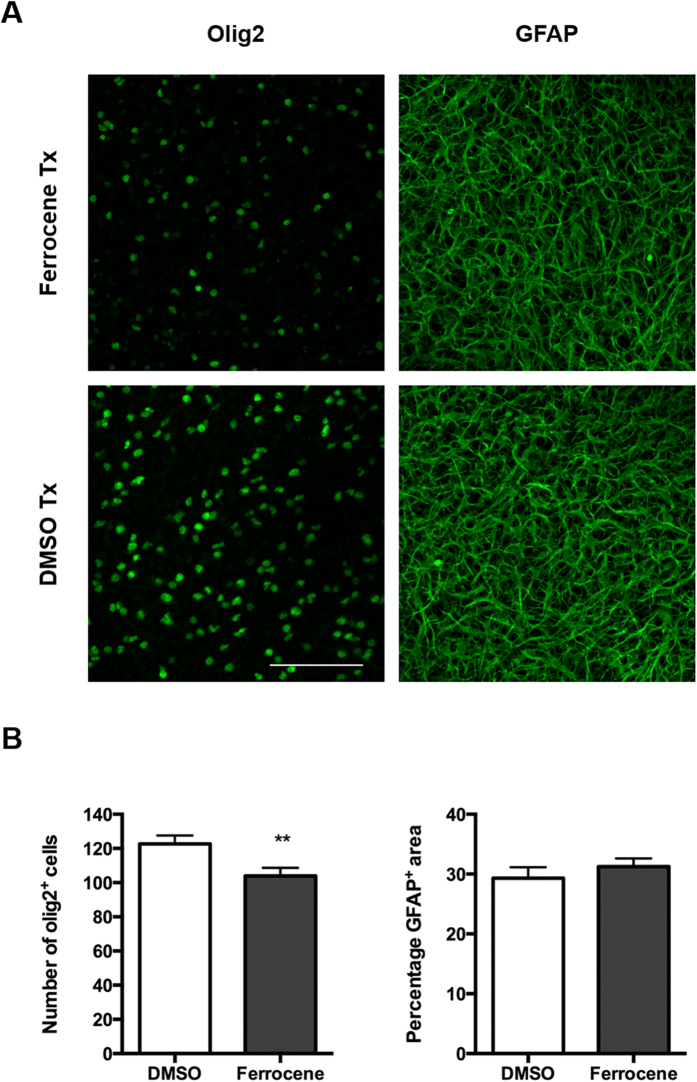
Effect of 1 μM ferrocene loading on the number and morphology of glial cells. Representative confocal maximum intensity projection images of (**A**) oligodendrocytes and astrocytes in vehicle and iron-loaded slice cultures. (**B**) Quantification of the number of olig2-positive cells and the GFAP-positive area per field. Iron loading reduces the number of olig2-positive cells but has no effect of astrocyte morphology and number. *Denotes P < 0.05 compared with vehicle. Scale = 100 μm.

Conversely, there was a 15% reduction in the number of olig2-positive cells in iron-loaded slice cultures compared with vehicle (123 ± 4.840 and 104.6 ± 4.916, respectively; Figs 6A,B & S3). Meanwhile, GFAP-positive astrocytes were unaffected by iron loading. There was no change in the area of GFAP-positive staining between treatment groups (Fig. 6A,B).

## Discussion

Organotypic cultures have demonstrated great potential as models of pathology in neurological disease, such as demyelination in Multiple Sclerosis^23^, amyloid deposition in Alzheimer’s disease^24^ and degeneration of dopaminergic neurons in Parkinson’s disease^25^. However, to date, these *ex vivo* cultures have not been realized as a platform to study iron handling in the CNS. Consequently, this present work sought to establish and optimise a novel model of iron loading using hippocampal slice-based methodology to mimic the iron deposition and aberrant distribution that can occur in neurodegenerative disease.

After demonstrating that the level of iron in control cultured hippocampal slices is representative of endogenous iron content in the age-matched hippocampus *in vivo*, we showed that in the 10–11-day-old rat brain, hippocampal iron levels are the same as those in the cerebellum and olfactory bulb and are half those in the cortex, brainstem and midbrain. This suggests, that in general terms, a hippocampus cultured slice platform is a useful starting point for studying iron regulation in the brain especially given that the hippocampus in itself is a reproducible and robust region for generating slice cultures. Furthermore, the iron content found in *in vivo* hippocampus and cortex in our study is on a par with values reported by Focht *et al.*^26^.

In the rat hippocampus *in vivo*, we documented a significant drop in iron content during development. This decrease in iron content in the hippocampus is in agreement with reductions reported by others in neonatal cortex, cerebellum and midbrain^27^.

Next, we found that 10 μM ferrocene caused maximal incorporation of iron into cultured hippocampus slices, when compared to FAS or FC, with a consequent modest reduction in slice viability. In a dose-response follow-on study, 1 μM ferrocene was discovered to be equally potent at causing iron overload, as both 1 and 10 μM ferrocene caused significant increases in iron content. Surprisingly, 100 μM ferrocene, was not effective in inducing the accumulation of iron.

The superior ability of ferrocene to cause iron overload may be due to its lipophilic structure (supplementary Fig. S4), which make it membrane-permeable and therefore able to initially bypass the rigorous iron homeostasis system that acts to keep iron levels under tight control. Previous studies have shown that iron is released from the ferrocene nucleus by enzymatic hydroxylation^28^,^29^,^30^,^31^. The resultant hydroxylated metabolite of ferrocene, which is unstable and oxygen dependent, undergoes spontaneous decomposition and releases solvated iron atoms that are available for normal physiological processing and metabolism^28^,^29^,^30^,^31^. On the other hand, ferrous and ferric iron (supplementary Fig. 4), are not lipophilic and must be taken up via DMT-1 and transferrin receptor, respectively. Iron loading from these reagents is therefore immediately subjected to the iron homeostasis machinery, which acts to down-regulate DMT1 and the transferrin receptor-mediated uptake of iron under conditions of excess iron^5^,^20^.

The degree of iron accumulation in our iron-loaded organotypic cultures is on a par with the increase in iron concentration detected in the post-mortem brain of people suffering from neurodegenerative disease. For example, iron content is elevated by approximately 1.5-fold in the AD hippocampus, 1.6-fold in MS grey matter structures and periplaque white matter, and 1.6-fold in PD substania nigral tissue, when compared with control brain^3^,^32^,^33^,^34^. Also, a similar increase in the iron levels in subcortical white matter of healthy people with aging has been reported^3^.

Several groups have reported similar increases in iron-loaded CNS monocultures. Different studies using primary astrocytes and microglia exposed to 10–33 μM FC or FAS for treatment periods ranging from 8–24 h reported a 2-fold increase^9^,^35^,^36^. Similarly, 20 μM TMPH-ferrocene 24 h exposure led to a 1.2-fold increase in oligodendrocyte precursor cells (OPCs)^37^. However, Bishop *et al.* demonstrated much higher 57- 35- and 19-fold increases in microglia, astrocytes and neurons, respectively, after 24 h incubation with 33 μM FAC^6^. While these large increases in iron content are impressive, the magnitude of iron accumulation in our model more closely matches that seen in human brain disorders.

In our iron-loaded slice cultures, there is an apparent increase and possible redistribution of ferritin expression in glial cells when compared to vehicle. We more frequently observed ferritin in olig2-positive cells in the iron-loaded slice cultures compared with vehicle. Furthermore, in an unbiased quantification of oligodendrocyte lineage cells using ImageJ, we demonstrated a significant 15% reduction in olig2-positive cells, suggesting that the lower level of slice viability detected could be a result of oligodendrocyte-lineage cell death. It is also possible that the loss of olig2-positive cells could be attributable to differentiation of early progenitors into other cell types, in response to iron-loading. However, at this stage of development in the hippocampus it is not expected that many of these multipotent cells would be present. Also, such a degree of differentiation would result in measurable increases in other cell types, which were not seen, suggesting that this change is largely due to loss of cells committed to the oligodendrocyte cell lineage. This is the first time, to our knowledge, that an iron-induced loss of oligodendrocytes from cultured hippocampal slices has been documented. Our data is consistent with reduced numbers of oligodendrocytes that has been previously reported in active MS lesions in post-mortem human tissue^3^. In that particular study, the loss of oligodendrocytes was accompanied by an up-regulation of iron-exporting ferroxidases and extracellular accumulation of iron (so-called iron liberation) followed by uptake of iron into microglia and macrophages. This is of interest since, in our brain slice model, we have observed seemingly increased ferritin staining in microglial cells following ferrocene treatment.

Iron overload in our model also impacted the microglial population, as we observed a modest increase in ferritin-positive microglia, which is in line with the reported 1.65-fold increase in iron influx in 20 μM ferric chloride-treated cultured primary microglia^35^, but is much less than the 57-fold increase in iron content in primary microglia exposed to 33 μM ferric ammonium citrate reported by Bishop *et al.*^6^. Ferrocene treatment also caused a significant increase in the number of microglia. We hypothesise that in a slice culture system proliferation is the only feasible source for the increased number of microglia cells. Hydrogen peroxide, which is produced by the oxidation of iron in ferritin^38^,^39^, has been described an important regulator of microglial proliferation^40^,^41^and might be responsible for the increase in microglia cells that we detected in our iron-loaded slice cultures.

When the morphology of microglia following exposure to ferrocene was examined, there was an obvious change that appeared to be consistent with activation^42^,^43^. This observation was confirmed using an objective Image J-based skeleton analysis^44^ of images from over 200 fields. The significant reduction in microglial ‘end-points’, when vehicle- and ferrocene-treated cultures were compared, meant that some microglia were in an iron-induced activated state. This data is consistent with reports that ferritin positivity and iron accumulation are associated with activated and dystrophic microglia in aged brains, as well as in brains from individuals who had AD, MS or Huntington’s disease^3^,^43^,^45^,^46^.

Astrocytes have previously been reported to accumulate large amounts of iron without any compromise in cell viability^6^,^8^,^10^,^36^. They respond somewhat similarly in our *ex vivo* slice culture model of iron-loading. On the one hand, we demonstrate only modest ferritin expression in iron-treated astrocytes, which is undetectable in control slices. On the other, in agreement with the previous reports regarding the robustness of astrocytes when it comes to iron toxicity, we do not show any difference in astrocyte morphology or number between iron- and vehicle-loaded slices. This implies that astrocytes, unlike oligodendrocytes, do not contribute towards the 1.6-fold increase in LDH release in iron-loaded cultures. It might also partially explain the apparently contradictory MTT and LDH assay results, since the metabolic activity of the plentiful astrocyte population in the slices may have obscured the reduced metabolism in other cell types.

Overall, in a study that is the first, to our knowledge, to characterise postnatal hippocampal iron content in any detail, we have demonstrated that the levels of iron in untreated cultured hippocampal slices replicate those of age-matched *in vivo* hippocampus. We also show, for the first time, that iron-loading slice cultures with ferrocene produces a maximal increase in iron content when compared to other commonly used iron reagents. The accumulation of iron was accompanied by increased ferritin in glia, mild toxicity, leading to oligodendrocyte loss, and microglia activation and proliferation. No appreciable effects were seen with astrocytes.

We believe that use of hippocampal slice-based methodology to produce a novel model of *ex vivo* iron accumulation is a significant advance on the mono- or dual-culture-based approaches that have been used up to now and that it will be an invaluable tool for researchers attempting to alleviate suffering caused by iron overload in the human brain.

## Materials and Methods

### Animals

Sprague–Dawley rats were obtained from Charles River Laboratories. All procedures were carried out in accordance with European directive 2010/63/EU and were approved by the Animal Care Research Ethics Committee of NUI Galway.

### Slice Culture Preparation and Maintenance

Organotypic hippocampal cultures were prepared from P10-11 male/female rat pups according to the methodology of Muller *et al.*^47^. Briefly, three 400 μm transverse hippocampal slices were cultured on Millicell culture inserts (Merck Millipore) at 34 °C in 5% CO_2_ for 10 DIV (days *in vitro*) prior to experimental treatment. The culture medium was composed of 50% MEM, 25% HIHS (heat-inactivated horse serum), 25% HBSS (Hank’s balanced salt solution), 28 mM D-glucose, 2 mM L-glutamine, 0.5 mM L-ascorbate, 63 μg/ml N-acetyl cysteine, 100 U/ml penicillin and 100 μg/ml streptomycin. For serum-free culture media, 25% HIHS was replaced with 25% MEM Eagle.

### Iron Loading

The following iron reagents were used: ferric citrate, ferrocene and ferrous ammonium sulphate (Sigma). These reagents (see supplementary Fig. S4) were chosen based on a literature search of previous iron loading studies and in order to encompass both ferric and ferrous forms of iron and also as a ‘disguised form’ of iron (i.e. one that bypasses import molecules) as ferrocene (supplementary Fig. S4)^30^,^35^,^36^,^48^,^49^,^50^. Previous studies have shown that iron is released from ferrocene and its derivatives in various biological systems and undergoes normal physiological processing and metabolism^31^,^51^.

Iron loading was carried out in serum-free free conditions. FC and FAS were reconstituted in dH_2_O and ferrocene in DMSO. Cultures were washed once with pre-warmed serum-free medium and then incubated in this for 12 h prior to experimental treatment. Cultures were loaded with iron reagent or its respective vehicle for 12 h in serum-free medium. FC and ferrocene were stored as 5 mM stock solutions and kept light-protected at room temperature (RT). FAS was freshly prepared before use. Cultures and media were processed as indicated below for each experiment.

### Iron Content

The total amount of iron present (i.e. both ferrous and ferric) in the slice cultures and tissue dissectates was determined using a ferrozine-based colorimetric method^36^. Briefly, slices were solubilised in NaOH (220 μl at 50 mM) and then sonicated (Branson digital sonifier; 10% amplitude, 1 pulse per s for 10 s once for slice cultures and three times for dissectates). Bound iron was released using freshly prepared acidic potassium permanganate at 60 °C for 2 h and detected after 30 min incubation with 60 μl iron detection reagent (6.5 mM ferrozine, 6.5 mM neocuproine, 2.5 M ammonium acetate, and 1 M ascorbic acid) by reading the absorbance of the ferrozine-iron complex at 550 nm in 250 μl reaction mixtures in wells of a 96-well plate^36^. Iron content was standardized to the protein content of slice cultures or tissue, as quantified by a Bradford assay.[Table t1]

### Cell Viability

Culture viability was determined by measuring the activity of lactate dehydrogenase (LDH) released into the culture medium and by quantifying metabolic activity^52^. The decrease in NADH absorbance, an index of LDH activity, was monitored at 340 nm for up to 10 min at RT. Maximal LDH release (i.e. maximal cell death) was defined as the level of LDH present in supernatant following complete lysis with 0.5% Triton X-100. The metabolic activity of cultures was assessed using an MTT [3-(4,5-Dimethylthiazol-2-yl)-2,5-Diphenyltetrazolium Bromide] assay in which the yellow tetrazolium salt is reduced by the mitochondria of viable cells into purple formazan crystals. Briefly, cultures were incubated for 30 min in serum-free medium containing MTT at a final concentration of 0.5 mg/ml. The formazan crystals were subsequently dissolved using a DMSO solution and the absorbance was then read at 595 nm.

### Real-time PCR

RNA was isolated from slice cultures using TRIreagent as per the manufacturer’s instructions and was quantified using a Nanodrop ND-100 spectrophotometer. After DNAse treatment, RNA was reverse transcribed using Superscript II Reverse Transcriptase and random primers. Gene expression was analysed by real time PCR using Fast SYBR Green Master Mix in the StepOnePlus Real-Time PCR System (Applied Biosystems) using specific primers for ferritin light and heavy chains (FTL and FTH) (see Table 1). The results were quantified using the ΔΔCT method following standardization relative to the internal control.

### Immunohistochemistry

Slices were fixed in 4% paraformaldehyde (PFA) for 30 min followed by three washes in PBS and storage at 4 °C in PBS containing 0.1% NaNO_3_. In order to minimise reagent volume, membrane around the slices was trimmed using a scalpel allowing incubations to take place in a 24–well plate. Slices that were still attached to membranes were blocked and permeabilised for 1 h with 10% normal goat serum (NGS; NGS was replaced with 10% horse serum when using the Iba1 goat antibody) and Triton X-100 (0.4%) in PBS. Subsequently, the slices were incubated with primary antibody (for single and dual labelling) in NGS (2.5%) Triton X-100 (0.1%) in PBS (or 2.5% horse serum for IBA1) for 48 h at 4 °C and then washed three times for 15 min before incubation with the appropriate secondary antibody at 4 °C overnight (see Table 2). Slice cultures were mounted in Vectashield containing diamino-2-phenylindole (DAPI) to allow visualisation of nuclei. Negative “no primary antibody” controls were included. Slides were stored in the dark at 4 °C until imaged.

Ferritin has previously been used as a sensitive surrogate marker for cytosolic iron accumulation because iron immunochemistry is not readily compatible with immunostaining for cell-specific markers^53^,^54^. The ferritin antibody (F5012, Sigma) used is mostly specific for ferritin light chain but ferritin heavy chain staining has also been described^3^,^54^,^55^. Given that neurons have previously been described predominantly to contain the heavy subunit whereas microglia, astrocytes and oligodendrocytes contain the light subunit^27^,^56^,^57^ this antibody is mostly useful as a surrogate marker of iron accumulation in glia rather than neuronal cells.

### Image Analysis

All samples were imaged on a laser scanning confocal microscope (Olympus Fluoview 1000), at 40 times magnification (oil immersion, numerical aperture of 1.3).

Due to morphological differences and the appearance of stained cells using different cell-specific antibodies, images of each cell type were captured and analysed using different image analysis paradigms, in NIH Image J software, as detailed below.

To determine the activation status and number of microglia, six representative fields (15 μm z-stack with 1 μm intervals) were acquired per slice i.e. 36 per treatment group for a combined total of 216 images from 3 independent experiments. Based on the method of Morrison *et al.*^44^, a custom macro was used to generate a maximum intensity projection image, subtract background using a rolling ball of 500 pixels and despeckle image (Fig. S1). Once the image was binarised, the skeleton was extracted using the *Skelentonise 2D/3D* plugin that iteratively erodes particles to generate centrelines that are a pixel wide (i.e. a skeleton) while maintaining particle connectivity. At this point, the number of skeletons could be equated with microglial cell number.

The *AnalyzeSkeleton* plugin (http://imagejdocu.tudor.lu/) was then applied to the skeletonised images to determine the number of endpoints per frame (subsequently normalised to the number of skeletons per frame. The number of endpoints is a measure of microglia branching complexity and loss of complexity indicates activation^42^,^43^,^44^.

In order to quantify changes in the oligodendrocyte population, seven fields (10 μm z-stack with 1 μm intervals) were acquired systematically throughout slice i.e. 42 images per treatment group, generating a combined total of 252 images from 3 independent experiment. Olig2-positive cells were counted using a custom macro (see Fig. S2 for details). Briefly, a maximum intensity projection image was generated for each z-stack, background was subtracted using a rolling ball of 50 pixels and thresholded using Otsu’s method^58^ to produce a 8-bit binary image where the pixels that exceed this threshold were converted to a value of 255 (black), while pixels below this threshold were converted to a value of 0 (white). The watershed segmentation algorithm was then used, which separates clusters of nuclei that are very close together, or touching, into individual cells. Finally, using the *Analyse Particles* function, the total number of particles with a size greater than 20 μm^2^ was determined.

To determine if astrocytes were affected by exposure to iron, slices were stained using GFAP antibody and the area of GFAP-positive staining was quantified. Five representative fields (5 μm z-stack with 1 μm intervals) were acquired per slice culture i.e. 20 fields over 4 slices per treatment, to generate a total of 120 images from 3 independent experiments. The GFAP-positive area was determined by generating a maximum intensity projection image for each z-stack, subtracting background with a rolling ball of 50 pixels and applying Bernsen threshold^58^, with a radius of 15 μm. Then, using the *Analyse Particles* function, the total area was determined. This area was expressed as percentage of the area of the total field.

The authors have adopted well established robust image analysis protocols to quantify the various cell types, these methods are compatible with automatization of large serial studies while allowing direct comparison with earlier studies. We are cognisant of the advantages of alternative design based stereological enumeration methods^59^,^60^.

### Statistical analysis

Data was analysed using Prism 6 (GraphPad Software). All measurements were expressed as mean  ±  standard error of the mean (SEM). All experiments were performed on three independent wells containing three slices unless otherwise specified. Normality was assessed using the Shapiro-Wilk test. Statistical analyses were carried out using Student’s t-test, Mann-Whitney test or ANOVA with Student Neumann Keuls post-test, as appropriate. Differences were considered statistically significant if P < 0.05.

## Additional Information

**How to cite this article**: Healy, S. *et al.* Significant glial alterations in response to iron loading in a novel organotypic hippocampal slice culture model. *Sci. Rep.* **6**, 36410; doi: 10.1038/srep36410 (2016).

**Publisher’s note**: Springer Nature remains neutral with regard to jurisdictional claims in published maps and institutional affiliations.

## Supplementary Material

Supplementary Information

## Figures and Tables

**Table 1 t1:** Primer sequences used for qPCR.

Gene	Accession	Forward	Reverse
FTL	NM_022500.4	CTCCTCAAGTTGCAGAACGAAC	GTTTTACCCCACTCATCTTG
FTH	NM_012848.2	ATCCCCACTTATGTGACTTC	CTTGTCAAAGAGATATTCTGCC
B-actin	NM_031144.3	CACACTGTGCCCATCTATGA	CCATCTCTTGCTCGAAGTCT
18s	M11188.1	AATCAGTTATGGTTCCTTTGTCG	GCTCTAGAATTACCACAGTTATCCAA

Abbreviations: FTL, ferritin light chain; FTH, ferritin heavy chain.

**Table 2:** Antibodies used for immunohistochemistry.

Name	Host/Isotype	Supplier	Catalogue number	Dilution
Olig2	Mouse mAb*	Millipore	MABN50	1:100
GFAP	Rabbit pAb	Dako	Z0334	1:1000
GFAP	Mouse mAb	Sigma	G3893	1:500
NeuN	Mouse mAb	Millipore	MAB377	1:1000
SMI-32	Mouse mAb	Abcam	AB28029	1:1000
Iba1	Goat pAb	Novus Biologicals	NB100-1028	1:100
Iba1	Rabbit pAb	Wako	091–10741	1:1000
Ferritin	Rabbit pAb	Sigma	F5012	1:200
Alexafluor anti-mouse 488	Goat pAb	Invitrogen	A11001	1:1000
Alexafluor anti-rabbit 594	Goat pAb	Invitrogen	A11037	1:1000
Alexafluor anti-goat 488	Donkey pAb	Invitrogen	A-11055	1:1000

^*^Abbreviations: mAb = monoclonal antibody, pAb = polyclonal antibody.
