# Supplementary material for: Significant glial alterations in response to iron loading in a novel organotypic hippocampal slice culture model
Source: Sci Rep. 2016 Nov 3;6:36410. doi: 10.1038/srep36410 (PMC5093415; doi:10.1038/srep36410)
Supplement: Supplementary Information [file srep36410-s1.pdf]

Supplementary material

Significant glial alterations in response to iron loading in a novel organotypic hippocampal slice culture model

Sinead Healy[1], Jill McMahon[1], Peter Owens[2], Una FitzGerald[1]

1. Galway Neuroscience Centre, National University of Ireland, Galway
2. Centre for Microscopy and Imaging, National University of Ireland, Galway, Ireland.

Mailing address:
Biomedical Sciences, Newcastle Road, Dangan, NUI Galway, Galway, IRELAND
Email:
una.fitzgerald@nuigalway.ie (corresponding author)

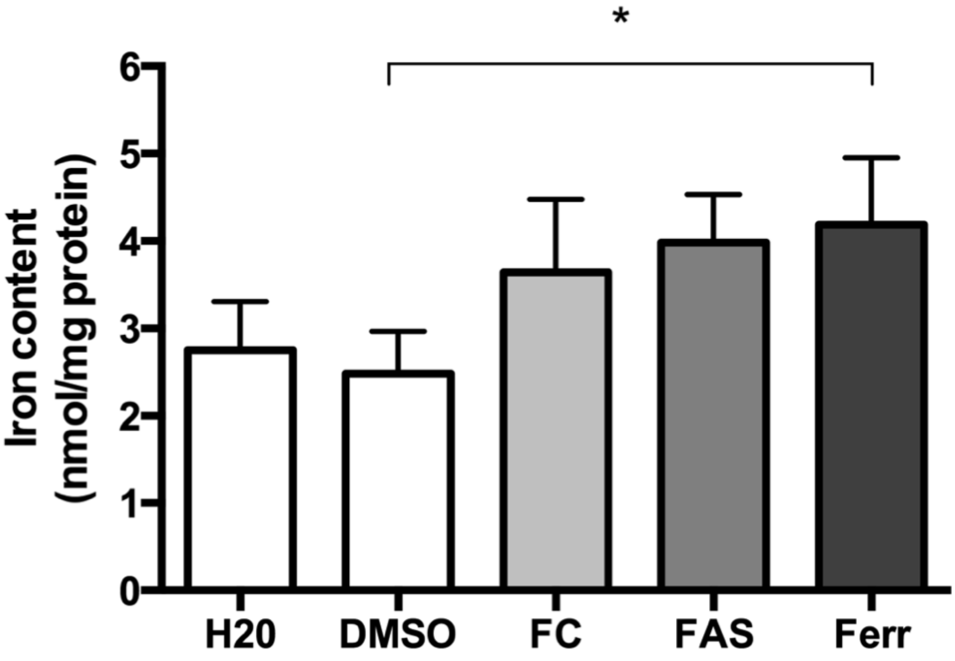

Figure S1. Iron reagent comparison. Cultures were treated for 12 h with 1 µM ferrocene, ferrous ammonium sulphate (FAS) or ferric citrate (FC). Ferrocene produced a significant 1.7-fold increase in iron content compared with DMSO. *$P<0.05$, ** $P<0.01$ compared with vehicle.

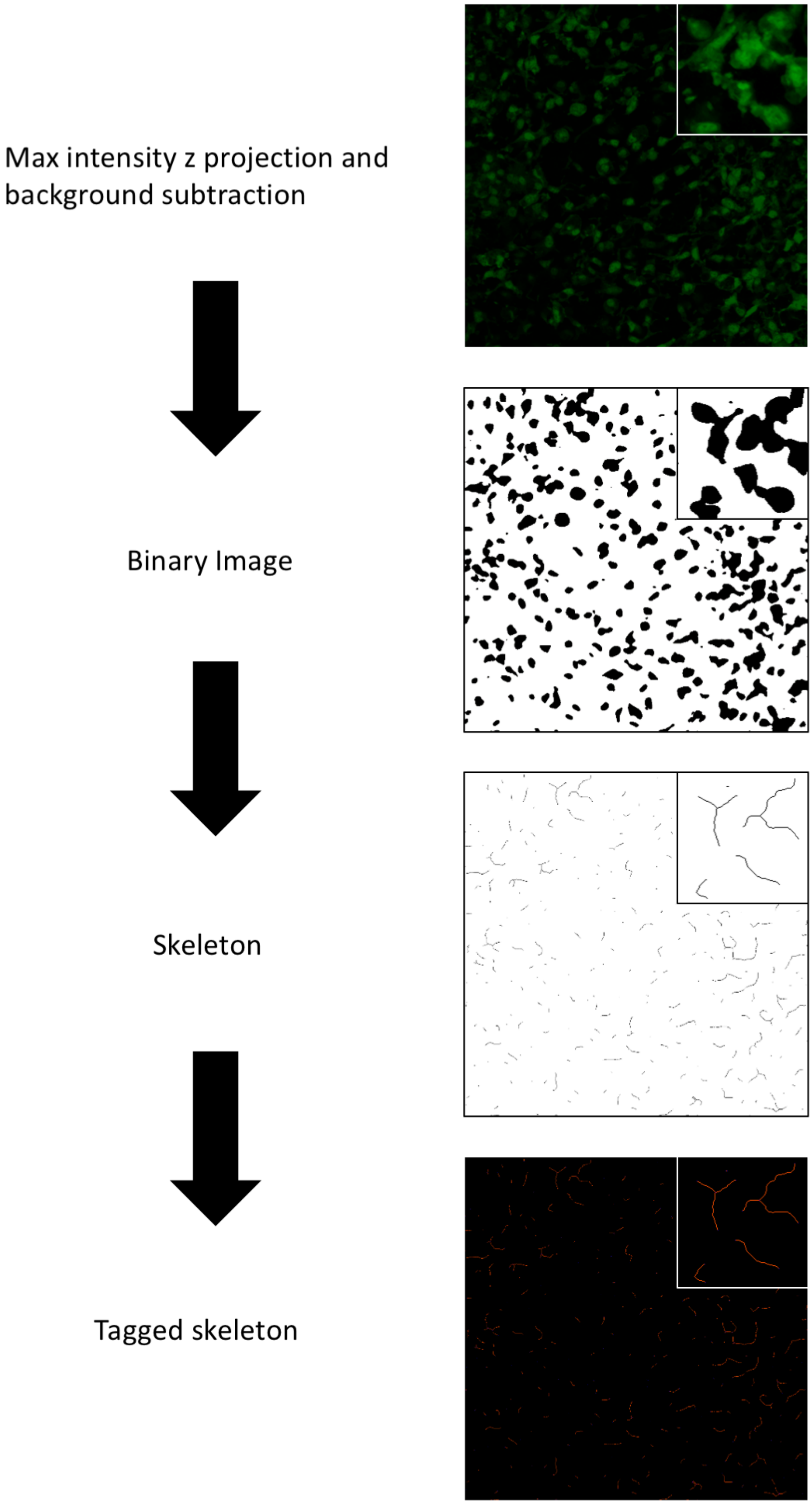

Figure S2. Image processing and quantification for microglia. Maximum intensity projections of confocal z-stacks were converted to binary images and then skeletonised to perform skeleton analysis. Custom macros were designed to automate these tasks in Image J

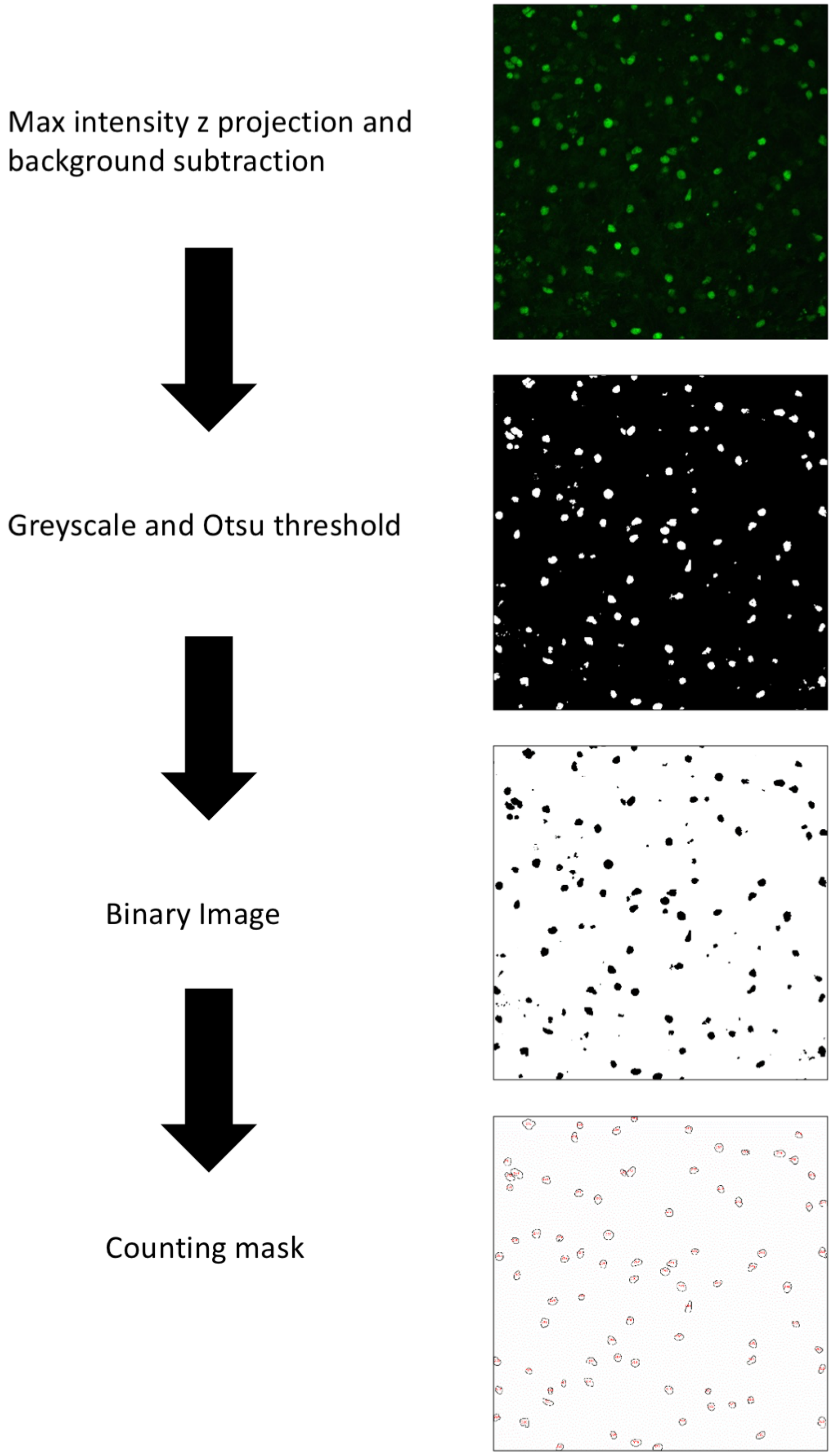

Figure S3. Image processing and quantification for oligodendrocytes. Maximum intensity projections of confocal z-stacks were generated, background was subtracted, image was converted to greyscale, thresholded using Otsu's method, converted to binary and then counted

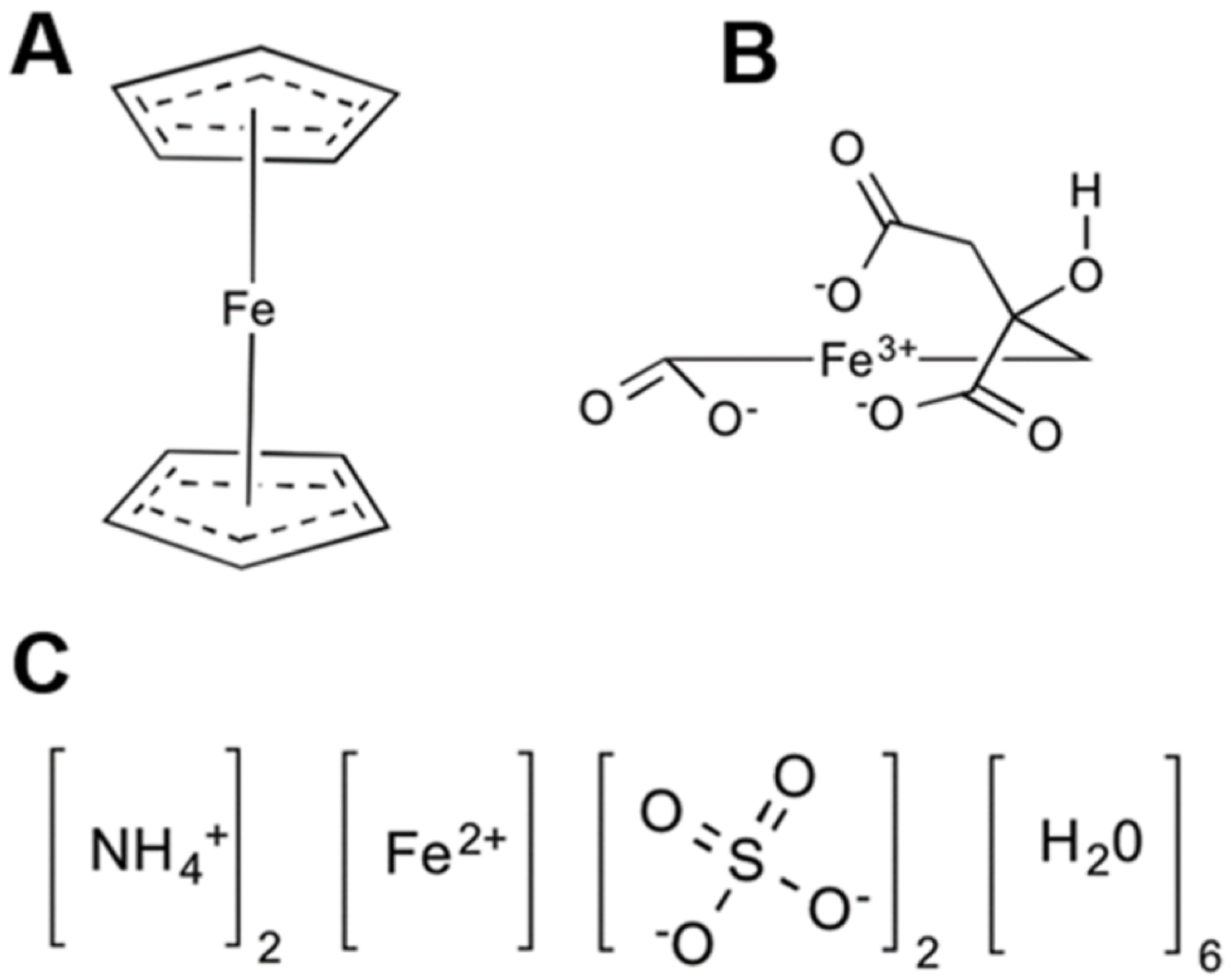

Figure S4. Chemical Structure of iron reagents used in this study. (A) Ferrocene (B) Ferric citrate and (C) Ferrous ammonium sulphate. The double-ring structure in ferrocene makes in highly hydrophobic and membrane-permeable. Ferric citrate and ferrous ammonium sulphate can only gain entry to the cell via transferrin receptor or DMT1, respectively.
